# Old Remedies for Epilepsy: Avicenna’s Medicine

**Published:** 2012-03-01

**Authors:** A A Asadi-Pooya, A R Nikseresht, E Yaghoubi

**Affiliations:** 1 Research Center for Traditional Medicine and History of Medicine, Shiraz Medical School, Shiraz University of Medical Sciences, Shiraz, Iran,; 2 Department of Neurology, Thomas Jefferson University, Philadelphia, USA; 3 Department of Neurology, Shiraz University of Medical Sciences, Shiraz, Iran; 4 Immunology Research Center, Shiraz University of Medical Sciences, Shiraz, Iran

**Keywords:** Avicenna, Epilepsy, Treatment

## Abstract

**Background:**

The history of epilepsy and its treatments dates back to at least 4 millennia. Avicenna, c. 980 AD in Bukhara, Khorasan–1037 in Hamedan was a Persian-speaking Iranian physician, who has many recommendations and suggested various therapies for epilepsy in his book, The Canon of Medicine.

**Methods:**

We first reviewed the most important ancient treatments for epilepsy mentioned by Avicenna and considered those as the key words for our next step. Then, we made a literature search (medline and scopus) with those key words to find out new scientific findings in modern medicine about the Avicenna’s suggestions.

**Results:**

Among the Avicenna’s recommended therapies for epilepsy, only Rue has been tested for anticonvulsant activities in modern medicine. Interestingly, it had a dose dependent anticonvulsant effect.

**Conclusion:**

It is worthwhile to consider the Avicenna’s recommended therapies for epilepsy and to design future scientific studies based on his suggestions.

## Introduction

Epilepsy ranks among the most common chronic neurological disorders. The history of epilepsy and its treatments dates back to at least four millennia, to the ancient civilizations of the Middles East. Ancient physicians contributed an enormous body of knowledge about seizures, their etiology, their manifestations, natural history and treatment. Past treatments have usually been empirical, reflecting the clinical observations of the ancient physicians, theological views or even superstitions. These treatments consisted of prescribed diets or living conditions, medicinal herbs, and occasionally surgery such as bloodletting or skull trephination.[1]

In the past decades, many new therapies have been introduced so that there are various options available to treat epilepsy. In spite of these recent advances, a large number of patients continue to have seizures and many experience adverse effects of treatment. As a result, there is an increasing interest in complementary therapies, including traditional or herbal medicine. In this study, we reviewed ancient treatments for epilepsy mentioned by Avicenna and compared them with the recent literature.

## Materials and Methods

In this article, we reviewed the most important ancient treatments for epilepsy mentioned by Avicenna, as the master in Iranian ancient medicine, and considered his suggestions as the key words for the next step of our study. Then, we made a literature search with those key words in Medline and Scopus to find out new scientific findings in modern medicine about the Avicenna’s recommendations, in order to make suggestions to design future scientific studies based on his suggested therapies for epilepsy.

## Results

Abu Ali al-Husayn ibn Abd Allah ibn Sina, c. 980 AD in Bukhara, Khorasan – 1037 AD in Hamedan, Iran, also known as Ibn Sina and commonly known in English by his Latinized name Avicenna, was a Persian-speaking Iranian, Muslim polymath and the foremost physician and islamic philosopher of his time. Avicenna wrote almost 450 treatises on a wide range of subjects, of which around 240 have survived and in particular, 40 of them concentrated on medicine. His most famous medical work is The Canon of Medicine, which was a standard medical text at many islamic and European universities until the early 18th century. Avicenna developed a medical system that combined his own personal experience with that of islamic medicine, the medical system of the Greek physician Galen, and ancient Persian, Mesopotamian and Indian medicine. Avicenna is regarded as a father of early modern medicine [proposals/Avicenna/Avicenna - Wikipedia, the free encyclopedia.mht].

Avicenna has many recommendations and suggested various therapies for patients with epilepsy in his book, The Canon of Medicine, some of these include:

### A. Beneficial Therapies for Epilepsy

1- Rue[2]: Rue (Ruta) is a genus of strongly scented evergreen subshrubs, in the family Rutaceae. In one study, the effects of an ethanol extract of the aerial parts of Ruta chalepensis on the central nervous system (CNS) were studied in mice. A crude extract was given systemically and its effects were tested on pentylenetetrazole (PTZ)-induced seizures, sodium pentobarbital-induced hypnosis, exploratory activity, anxiety and nociception. A delay in the onset of seizures and a dose-dependent suppression in the tonic phase and mortality induced by PTZ, a prolongation of the time of sodium pentobarbital-induced hypnosis, a significant attenuation in the anxiety-response and a reduction in the licking time and shaking behavior in the formalin-induced nociception test have been ob-served. The sedative-hypnotic potentiation, anxiolytic, anticonvulsant and antinociceptive effects suggested that Ruta chalepensis induces a depressant activity on the CNS.[3]

2- Chives[2]: Chives (Allium schoenoprasum) are the smallest species of the onion family. The medical properties of chives are similar to those of garlic. It containes many organosulfide compounds, vitamins A and C, and trace amounts of sulfur and iron. One study indicated that extracts from all plant organs exhibited antioxidant activity. The highest antioxidant activity was observed in the leaves.[4] Epidemiologic and laboratory studies suggested that allium vegetables and garlic constituents had antitumor effects.[5] The probable antiepileptic properties of this plant have never been tested in the modern medicine.

3- Savory[2]: Summer savory (Satureja hortensis) is the better known of the Savory species. This herb has traditionally been used in treatment of cardiovascular diseases and thrombosis. In one study, it was observed that in addition to alteration of cell adhesive properties, self aggregation and protein secretion of the treated platelets were also affected upon treatment with the crude methanol extract of this plant.[6] In another study, both the ethanolic extract and the essential oil of the plant reversed the oxidative damage to rat lymphocytes induced by hydrogen peroxide.[7] It also has antibacterial, antifungal, and antioxidant activities,[8] antinociceptive, anti inflammatory effects,[9] antispasmodic and anti-diarrheal properties.[10] Satureja hortensis has a strong inhibitory effect on aflatoxin production by A. parasiticus.[11] The probable antiepileptic properties of this plant have never been tested in the modern medicine.

4- Wormwood[2]: Artemisia arborescens (Sheeba in Arabic) is a very bitter herb indigenous to the Middle East. Thujone, camphor and chamazulene account for about 75% of its oil.[12] This plant has anti-viral activity against HSV-1 and HSV-2.[13] In one animal study, the aqueous extract of this plant caused a concentration dependent reduction in the amplitude of the phasic contractions and in the tone of the ileum.[14] This plant has been employed for the treatment of several diseases such as malaria, hepatitis, cancer, inflammation and infections sustained by fungi or bacteria.[13] The probable antiepileptic properties of this plant have never been tested in the modern medicine.

5- Dill[2]: Dill (Anethum graveolens) is a short-lived annual herb. This plant has significant lipid lowering effects.[15] Dill has some effects on the female reproductive system and can be used either as a regulatory agent of the menstrual cycle for women with irregular cycles or as an antifertility agent.[16] It also has antimicrobial activity[17][18] and significant mucosal protective and antisecretory effects in the gastric mucosa.[19] The probable antiepileptic properties of this plant have never been tested in the modern medicine.

6-Hyssop[2]: Hyssop (Hyssopus) is a genus of about 10-12 species of herbaceous or semi-woody plants in the family Lamiaceae. Hyssop oil is an important food additive and herbal medicine.[20] Hyssop is a source of natural antioxidants and its extract may inhibit the digestion of complex carbohydrates, but not that of absorbable monosaccharide, and might be a useful supplemental food for hyperglycemia.[21] Hyssop officinalis extracts exhibit strong anti-HIV activity, and may be useful in the treatment of patients with AIDS.[22] The probable antiepileptic properties of this plant have never been tested in the modern medicine.

7- Truffle, wild boar meat, phelebotomy, and balanced diet[2] are among other remedies for epilepsy recommended by Avicenna. The probable antiepileptic properties of these have never been tested in the modern medicine era.

### B. Avicenna's Recommendations with Regard to Seizure Precipitating Factors

Seizures often occur spontaneously without apparent inciting cause in most individuals, but many patients believe that their seizures are precipitated by external or internal stimuli. Some of these precipitating factors have been well defined in the recent literature. These include sleep deprivation, excess alcohol intake, premature awakening, psychological stress, prolonged fasting, physical exhaustion, and photic stimulation.[23] Knowing which factors might precipitate seizures could help gain a better understanding of how some seizures might be avoided in certain individuals. Avicenna has suggested that patients with epilepsy should avoid precipitating factors for seizures, some of those include 1- Alcohol, 2- Overfeeding, 3- Sleep deprivation and 4- Oversleeping.

Overfeeding and oversleeping have not been mentioned and studied as probable seizure precipitating factors in the recent literature.

## Conclusions and Recommendations for Future Studies

Among the Avicenna’s recommended therapies for epilepsy, only Rue has been tested for anticonvulsant activities in modern medicine. Interestingly, it had a dose dependent anticonvulsant effect. Indeed, nature has served as a rich source of drugs for centuries and an impressive number of modern drugs have been derived from natural sources, notably of plant origin. We believe it is worthwhile to consider the Avicenna’s recommended therapies for epilepsy and to design future scientific studies based on his suggested therapies for epilepsy, as he was a famous scientist and physician with extraordinary observations and experiences.
